# Genotoxicity of Microcystin-LR in *In Vitro* and *In Vivo* Experimental Models

**DOI:** 10.1155/2014/949521

**Published:** 2014-05-18

**Authors:** Elsa Dias, Henriqueta Louro, Miguel Pinto, Telma Santos, Susana Antunes, Paulo Pereira, Maria João Silva

**Affiliations:** ^1^Department of Environmental Health, National Institute of Health Dr. Ricardo Jorge, Avenida Padre Cruz, 1649-016 Lisbon, Portugal; ^2^Department of Human Genetics, National Institute of Health Dr. Ricardo Jorge, Avenida Padre Cruz, 1649-016 Lisbon, Portugal

## Abstract

Microcystin-LR (MCLR) is a cyanobacterial toxin known for its acute hepatotoxicity. Despite being recognized as tumour promoter, its genotoxicity is far from being completely clarified, particularly in organs other than liver. In this work, we used the comet and/or the micronucleus (MN) assays to study the genotoxicity of MCLR in kidney- (Vero-E6) and liver-derived (HepG2) cell lines and in blood cells from MCLR-exposed mice. MCLR treatment (5 and 20 *μ*M) caused a significant induction in the MN frequency in both cell lines and, interestingly, a similar positive effect was observed in mouse reticulocytes (37.5 *μ*g MCLR/kg, i.p. route). Moreover, the FISH-based analysis of the MN content (HepG2 cells) suggested that MCLR induces both chromosome breaks and loss. On the other hand, the comet assay results were negative in Vero-E6 cells and in mouse leukocytes, with the exception of a transient increase in the level of DNA damage 30 minutes after mice exposure. Overall, the present findings contributed to increase the weight of evidence in favour of MCLR genotoxicity, based on its capacity to induce permanent genetic damage either *in vitro* or *in vivo*. Moreover, they suggest a clastogenic and aneugenic mode of action that might underlie a carcinogenic effect.

## 1. Introduction

Microcystins are secondary metabolites of cyanobacteria occurring worldwide in freshwater resources and have been associated with episodes of human and animal acute liver toxicity [1, 2]. The liver specificity of microcystins has been attributed to the selective expression by hepatocytes of a family of membrane transporters, the organic anion polypeptide transporter (OATP) [3, 4], through which microcystins enter into the cells. The mechanism of acute hepatotoxicity is believed to be mediated by the inhibition of serine/threonine protein phosphatases 1 and 2A (PP1 and PP2A) [5] and the consequent induction of cytoskeletal proteins hyperphosphorylation leading to hepatocyte deformation, collapse of tissue organization, and necrosis [6]. Increasing evidences have demonstrated that microcystins might also target other organs such as kidney [7–9], intestine [10, 11], lungs [12], reproductive system [13], and brain [14], although the knowledge about its toxicity on these organs is very restricted. The establishment of a guideline for microcystins in drinking water [15] and the implementation of water quality surveillance programs have contributed to a decrease in the risk of acute intoxication by waterborne cyanotoxins [16].

However, microcystins are classified by the International Agency for Research on Cancer as possibly carcinogenic to humans [17]. Epidemiological studies have suggested an association between chronic exposure to low doses of these toxins through drinking water and an increase in primary hepatocellular [18, 19] and colorectal cancers [20]. Furthermore, two-stage rodent carcinogenesis assays have demonstrated that microcystin-LR (MCLR), the most abundant and studied microcystin variant, is involved in tumour promotion [21–23] while several* in vivo* and* in vitro* studies have pointed to a genotoxic activity of MCLR (revised in [24]), although this issue is not consensual.

Several reports have shown that MCLR induces DNA damage in liver cells* in vivo* [10, 25, 26] and in distinct cells types* in vitro* [25, 27–34], but the mechanism behind the observed DNA breakage is not clear and seems to be dose-dependent and cell-type dependent. To our knowledge, no MCLR-DNA adduct has been identified so far, suggesting an indirect mechanism for its genotoxicity. In fact, oxidative stress was proposed as a mechanism of MCLR-induced DNA damage [29–33]. Supporting this hypothesis, studies on liver cells have demonstrated that subcytotoxic doses of MCLR induce the formation of 8-oxo-deoxyguanosine (8-oxo-dG), a marker of oxidative DNA damage [35, 36]. On the contrary, some authors have attributed the MCLR-induced DNA lesions, measured by the comet assay, to endonucleolytic DNA degradation associated with apoptosis [27] or necrosis [25] rather than to genotoxic events. A permanent chromosome damaging effect has been additionally suggested for microcystins by studies that showed an induction of micronuclei (MN)* in vivo* [28] or* in vitro* [37]. In contrast, other authors reported no effect of MCLR on the micronucleus frequency in different cell models [38–41], in agreement with the negative results of the chromosome aberrations analysis [27, 42].

The controversy concerning MCLR genotoxicity probably arises from the analysis of distinct endpoints in different* in vivo* and* in vitro* biological models exposed to distinct microcystins sources (pure toxin or cyanobacterial extracts), hampering the establishment of a definitive conclusion about MCLR genotoxicity (reviewed in [24]).

Despite its toxicity, recent studies have suggested that MCLR might be exploited to be used as an anticancer agent [43, 44]. This possibility arises from the observation that some tumours overexpress OATPs comparatively to the corresponding normal tissues [44] and that MCLR, at subnanomolar concentrations, is a potent cytotoxic agent against OATP-transfected tumour cells [43].

Considering this risk/benefit duality of MCLR, the characterization of its genotoxicity has a twofold goal: either to evaluate the potential health hazard from continuous exposure to low doses from environmental sources or to evaluate the safety of MCLR considering their potential pharmacological applications.

The present study was aimed at contributing to the genotoxicity evaluation of MCLR* in vitro*, in two mammalian cell lines representative of liver and kidney (HepG2 and Vero-E6 cell lines, resp.) and* in vivo* in C57Bl/6 mice. In order to obtain the maximum information about MCLR genotoxicity from these experimental models, a combination of the micronucleus and the comet assays was selected. Such a combination covers different genetic endpoints, given that the DNA strand breaks and alkali-labile sites measured by the comet assay are primary DNA lesions with relevance for gene and chromosome mutation formation whereas micronuclei reflect chromosome abnormalities due to clastogenic and/or aneugenic events [45–47]. Moreover, gene mutations and numerical/structural chromosome changes are relevant for carcinogenesis and the cytokinesis-block micronucleus assay has been shown to have a predictive value for cancer risk [48]. In order to add some insights into MCLR's mode of action, we evaluated micronucleus content using the fluorescence* in situ* hybridization (FISH) coupled to the micronucleus assay.

## 2. Materials and Methods

### 2.1. Genotoxicity Assays in Vero-E6 and HepG2 Cell Lines

#### 2.1.1. Cell Lines and Reagents

The Vero-E6 (African green monkey,* Cercopithecus aethiops,* kidney epithelial cells) and HepG2 (human hepatocellular carcinoma) cell lines were obtained from the American Type Culture Collection (ATCC-CRL 1586) and German Collection of Microorganisms and Cell Cultures (DSMZ ACC 180), respectively. Vero-E6 cells were grown in Modified Eagle Medium (MEM) supplemented with 10% fetal bovine serum (FBS), 0.1 mM nonessential amino acids, and 1 mM sodium pyruvate. HepG2 cells were grown in RPMI 1640 w/Glutamax, containing 15% FBS. Both cell lines were maintained at 37°C, in a 5% CO_2_ humidified incubator. All culture media and supplements were purchased from Gibco-Invitrogen (Paisley, UK).

Microcystin-LR (CAS Number 101043-37-2) was purchased from Alexis/Enzo Life Sciences (Lausen, Switzerland) as a white solid film (purity ≥ 95%, by HPLC). A stock solution of MCLR (1 mM) was prepared by dissolving the toxin in cell culture medium or saline solution and kept at −20°C until use. Work solutions of 5 and 20 *μ*M of MCLR were prepared immediately before testing, by diluting the stock solution in cell culture medium or saline solution.

#### 2.1.2. Cytotoxicity (Neutral Red) Assay

The neutral red (NR) assay [49] was used to evaluate the cell lines viability after exposure to MCLR. This assay was conducted in 96-well plates containing 1 × 10^4^ viable cells/100 *μ*L of growth medium. Cells were exposed to 5 and 20 *μ*M of MCLR during 24 h. H_2_O_2_ (400 mM, 1 h) was included as positive control and untreated cells were the negative control. Three replicates were performed per treatment condition. After treatment, the exposure medium was replaced by fresh culture medium containing a NR solution (50 *μ*g/mL; Merck, Darmstadt, Germany) and incubated for 3 h. Cells were rinsed with PBS and the incorporated NR was extracted with an ethanol : acetic acid : water (50 : 1 : 49) solution. NR incorporation was quantified spectrophotometrically at 540 nm using a Multiscan Ascent spectrophotometer (Labsystems, Helsinki, Finland).

The relative cell viability, expressed as the percentage of viable cells, was estimated as the ratio between the mean absorbance of treated and control cells, assuming the mean absorbance of the negative control to represent 100% viable cells. The results are expressed as the mean value (±SE) of three independent experiments per treatment condition.

#### 2.1.3. Comet Assay

Vero-E6 cells were plated in 24-well plates, at a density of 5 × 10^4^ cells per well, and incubated at 37°C for 24 h to allow for cells adherence and growth. After 24 h, the culture medium was replaced by fresh medium containing 5, 10, and 20 *μ*M of MCLR. Cells were exposed, in duplicate cultures, for short (4 h) and long (24 h) periods. Simultaneously, positive (H_2_O_2_, 100 *μ*M for 30 min at the end of the exposure period) and negative (nontreated cells) controls were included. The comet assay was performed according to Pinto et al. [50]. Briefly, following exposure 5 × 10^4^ cells were mixed with 80 *μ*L of 1% low melting point agarose to prepare 2 gels on microscope slides previously covered with a 1% normal melting point agarose layer. Cells were lysed (2.5 M NaCl, 100 mM EDTA, 10 mM Tris, 1%* N-*lauroylsarcosine, 10% DMSO and 1% Triton X-100, pH 10) for at least 1 h at 4°C, and nucleoids were treated with FPG (kindly provided by Dr. A. R. Collins, University of Oslo, Norway) or buffer only (40 mM HEPES, 100 mM KCl, 0.5 mM EDTA, BSA 0.2 mg/mL, pH 8), for 30 min at 37°C. DNA was allowed to unwind in alkaline conditions (300 mM NaOH, 1 mM Na_2_EDTA·2H_2_O, pH > 13) for 40 min before subjecting to electrophoresis for 30 min at 0.7 V/cm. Finally, slides were rinsed with neutralization buffer (0.4 M Tris-HCL, pH 7.5) followed by dH_2_O, for 10 min each, stained with ethidium bromide (0.125 *μ*g/*μ*L), and analysed using a fluorescence microscope (Zeiss, Axioplan 2, Göttingen, Germany). Fifty randomly selected nucleoids per gel (i.e., 100 nucleoids per slide) were analysed with the Comet Imager 2.2 image analysis software (MetaSystems, GmbH, Altlussheim, Germany). The percentage of DNA in the comet tail was selected to measure DNA damage and the results represent the mean (±SE) of three independent experiments.

#### 2.1.4. Micronucleus Assay

Cells were seeded in 6-well plates at a density of 5 × 10^4^ viable cells per well. Following 24 h, the growth medium was replaced by fresh medium containing 5 or 20 *μ*M of MCLR and 1 h later cytochalasin B (6 *μ*g/mL Sigma-Aldrich) was added. For each experiment, negative (nontreated cells) and positive (MMC, 0.1 *μ*g/mL) controls were included. At 24 h after treatment, cells were trypsinized and harvested using a hypotonic treatment (0.075 M KCl; 3 min, at room temperature) followed by fixation with cold fixative (acetic acid : methanol, 1 : 3) and spread onto glass slides by cytocentrifugation (Shandon Cytospin, Themo Scientific, MA, USA) at 1200 rpm, 5 min. After being air-dried, slide preparations were stained in 4% Giemsa (Merck, Darmstadt, Germany) in phosphate buffer, pH 6.8, mounted with Entellan (Merck), and analysed using a light microscope (Zeiss, Axio Imager A2).

Three independent experiments were performed with Vero-E6 cells and two experiments were performed with HepG2 cells, using two replicate cultures per treatment condition. A total of 1000 cytokinesis-blocked cells (binucleated cells) per replica were scored for the presence of micronuclei (MN), using the criteria described by Fenech et al. [51]. In addition, the proportion of mono-, bi-, and multinucleated cells was calculated by scoring 1000 cells per treatment condition. The cytokinesis-block proliferation index (CBPI) was calculated by the formula: CBPI = (*M*1 + 2*M*2 + 3*M*3)/*N*, where *M*1-*M*2 represents the number of cells with 1-2 nuclei, *M*3 represents the number of cells with more than 2 nuclei, and *N* is the total number of scored cells [52, 53].

#### 2.1.5. Fluorescence* In Situ* Hybridization (FISH)

To determine whether MCLR-induced MN in HepG2 cells were originated from a clastogenic (centromere-negative, cm−) or aneugenic (centromere-positive, cm+) mechanism, the presence of centromeres inside the MN was investigated by the FISH assay using a biotin-labelled human pancentromeric probe (Cambio, Cambridge, UK). This analysis was not performed with the Vero-E6 cell line because of the unavailability of a green monkey pancentromeric probe.

HepG2 cells (treated with 20 *μ*M of MCLR) previously fixed and spread onto glass slides for micronucleus analysis were also used for the FISH assay, performed according to manufacturer instructions. Colcemid-treated (0.02 *μ*g/mL) and untreated cultures were used as positive and negative controls, respectively. Briefly, slides were washed in 2xSSC buffer, dehydrated through ethanol series, and allowed to dry. Cells were denatured with 70% formamide (in 2xSSC) at 70°C for 2 min and dehydrated through ethanol series. Hybridization with previously denatured DNA probe (10 min, 85°C) was performed overnight at 40°C in a humid chamber. Posthybridization washes were performed with 50% formamide in 2xSSC (5 min, 37°C) followed by 2xSSC (5 min, 37°C). The hybridization signals were revealed using an Avid-Cy3 antibody (1 hour at 40°C; Amersham, Uppsala, Sweden). Cells were counterstained with DAPI (0.5 *μ*g/mL) and finally mounted in Vectashield (Vector Laboratories, Burlingame, CA, USA). Slides were analysed under a fluorescence microscope (Zeiss, Axioplan 2) using filters for DAPI and Cy3 at a magnification of 630x. The number of cm+ and cm− MN was scored within 1000 binucleated cells.

### 2.2. Genotoxicity Assays* In Vivo* in Mice Blood Cells

#### 2.2.1. Animals, Toxin Administration, and Blood Sampling

Male mice from the C57Bl/6 strain, 9-10 weeks old, weighing approximately 20 g, were maintained, treated, and sacrificed at the National Institute of Health Dr. Ricardo Jorge according to the European Union directives. The room temperature was 21–23°C, the light/dark cycle was 12 h/12 h, and lab chow and water were supplied* ad libitum*.

Six mice were injected intraperitoneally (i.p.) with 37.5 *μ*g MCLR/kg of body weight (bw). The toxin was diluted from a stock solution of MCLR (1 mM) prepared in saline solution. Peripheral blood samples were collected before the treatment (0 h) and at several timepoints after treatment for the comet (0.5, 1, 24, and 48 h) and the micronucleus (48 and 72 h) assays. The negative control consisted of samples collected at 0 h, and positive control for both assays was ethylnitrosourea (ENU, 100 mg/kg bw; Sigma-Aldrich), administered i.p.

#### 2.2.2. Comet Assay in Mice White Blood Cells

Peripheral blood was collected from the mouse tail using a heparinised micropipette tip. A 5 *μ*L sample was diluted in 10 *μ*L of cold mincing solution (20 mM EDTA disodium, 10% DMSO, prepared in D-PBS Mg^2+^/Ca^2+^/phenol red free). Five *μ*L of this solution was mixed with 75 *μ*L of 0.7% low melting point agarose to prepare two gels per sample on a microscope slide previously covered with a 1% agarose layer. The comet assay was thereafter performed as described for cell lines.

#### 2.2.3. Micronucleus Assay in Mouse Reticulocytes

A peripheral blood sample collected from the mouse tail was dropped onto glass slides precoated with acridine orange (1 mg/mL) and covered with a coverslip. Prior to analysis, slides were maintained in a humidified chamber at 4°C. Mice micronucleated reticulocytes (MNRet) were scored in a total of 2000 reticulocytes (Ret) per animal, under fluorescence microscopy. The percentage of Ret was also examined as a measure of toxicity.

### 2.3. Statistical Analysis

Fisher's exact test (2-tailed) was used to determine whether MCLR induced a statistically significant difference in the frequency of micronucleated binucleated (MNBC) Vero-E6 and HepG2 cells as compared with the untreated control cultures. The same test was used to evaluate if MCLR induced a statistically significant change in the proportion of cm+/cm− micronuclei in HepG2 cells, compared to control cultures.

To determine whether MCLR induced a statistically significant different frequency of MNRet in relation to the control group, the two-tailed Student's* t*-test was applied.

Nonparametric Mann-Whitney *U* test was used to compare the percentage of tail DNA between treated and control mice groups, at several timepoints.

For all tests, the threshold of significance was *P* < 0.05.

## 3. Results

### 3.1. *In Vitro* Genotoxicity

**Figure 1:**
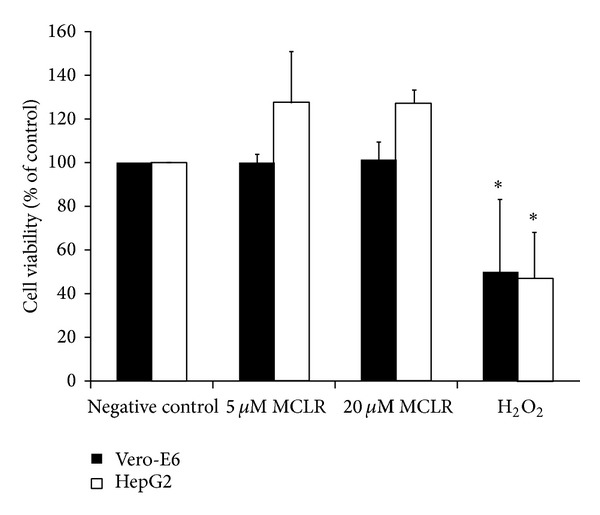
Viability of Vero-E6 and HepG2 cell lines exposed to MCLR (5 and 20 *μ*M, 24 h) and assessed by the NR assay. H_2_O_2_ (400 mM, 1 h) was used as a positive control. Results are expressed as the mean percentage of absorbance values relative to the negative control (±SD) from three independent experiments tested in triplicate. ∗represents a statistically significant difference between the treated and the control cells (*P* < 0.05).

As it is shown in Figure 1, none of the tested MCLR concentrations interfered with the viability of Vero-E6 and HepG2 cell lines. This confirms the absence of cytotoxic effects in the range of MCLR concentrations used in the genotoxicity assays.

**Figure 2:**
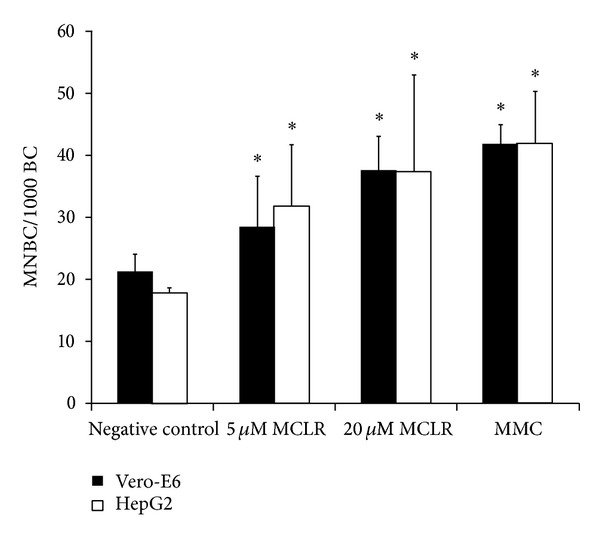
Micronucleus in cytokinesis-blocked Vero-E6 and HepG2 cells, following exposure to MCLR for 24 hours. Results are expressed as the mean frequency of micronucleated binucleated cells (MNBC) per 1000 binucleated cells (BC). Mean (±SD) were obtained from three (Vero-E6) or two (HepG2) independent experiments, using duplicate cultures. Mitomycin C (MMC, 0.1 *μ*g/mL, 24 h) was used as the positive control of the assay. ∗represents a statistically significant difference between the treated and the control cells (*P* < 0.05).

The data from the DNA damaging effect of MCLR in the Vero-E6 cell line as assessed by the comet assay and the oxidative DNA damage estimated by the FPG-modified comet assay are presented in Table 1. None of the tested MCLR concentrations (5, 10, and 20 *μ*M) increased significantly the level of DNA lesions in Vero-E6 cells after a 4- or 24-hour exposure period, as compared to controls. The genotoxicity of MCLR was further evaluated by the MN assay in cytokinesis-blocked Vero-E6 and HepG2 cells (Figure 2). In both cell lines the two tested MCLR concentrations (5 and 20 *μ*M) were able to significantly increase the frequency of MNBC, as compared to the negative control (Figure 2). Vero-E6 cells treatment with 5 *μ*M of MCLR produced a significant 1.3-fold increase in the frequency of MNBC (*P* = 0.012), whereas a 1.8-fold increase was observed in treated HepG2 cells (*P* < 0.0001). For the highest toxin concentration a significant 1.8-fold raise was observed for Vero-E6 cells (*P* < 0.0001) and a 2.1-fold raise was observed for HepG2 cells (*P* < 0.0001). For each treatment condition the proportion of mono-, bi-, and multinucleated cells was calculated to determine the CBPI that gives an estimate of cytotoxicity. The percentage of binucleated cells ranged between 45%–56% and 64%–72% in Vero-E6 and HepG2 cultures, respectively, and no differences were observed between MCLR-treated and untreated cells, independently of the dose. The percentages of binucleated cells are in agreement with values for optimal culture conditions [52, 54]. The estimated CBPI was 1.5-1.6 for Vero-E6 cells and 1.6-1.7 for HepG2 cells and was not affected by the MCLR treatment suggesting that the tested toxin concentrations do not interfere with the normal progression of cells through the cell cycle and confirming that they do not affect cell viability [55].

**Figure 3:**
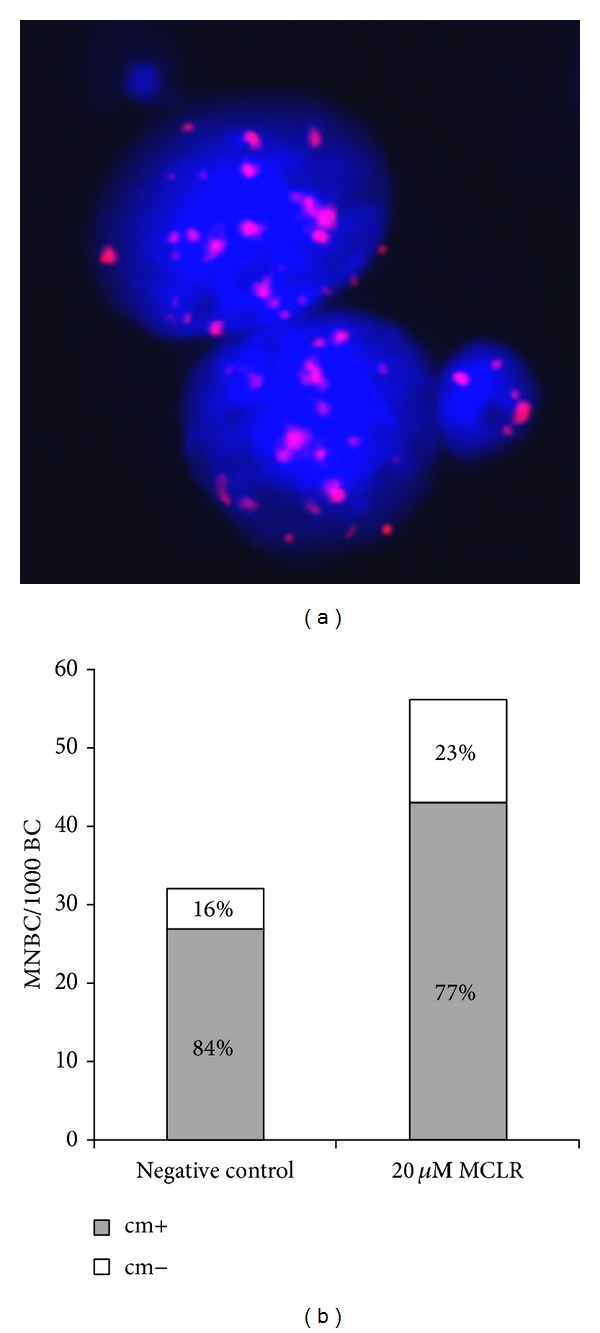
Characterization of centromere-positive (cm+) and centromere-negative (cm−) micronuclei in untreated and MCLR-treated HepG2 cells (20 *μ*M, 24 h) by FISH analysis using a human pancentromeric probe. (a) A binucleated cell with a cm+ (2 hybridization red signals) and a cm− MN, following MCLR treatment. (b) Absolute and relative frequencies of cm+ and cm− MN per 1000 binucleated (BC) HepG2 cells.

The analysis of MN content (Figure 3) showed that the relative proportion of cm+ and cm− MN did not differ significantly between MCLR-treated and untreated HepG2 cells (*P* = 0.584), although the induction of cm− (2.6-fold) was more pronounced than the induction of cm+ (1.6-fold) MN.

### 3.2. *In Vivo* Genotoxicity

**Figure 4:**
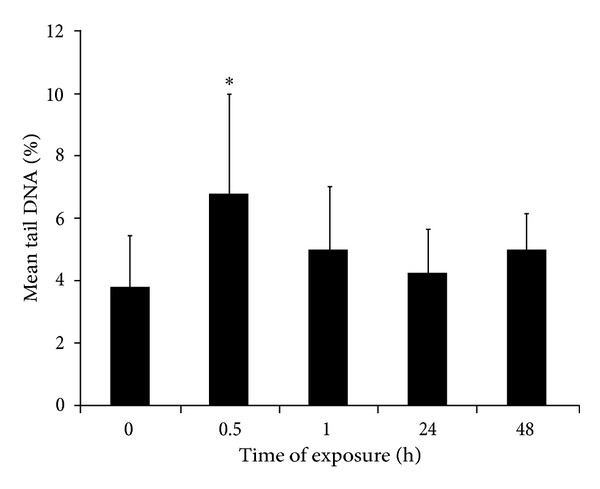
DNA damage evaluated by the comet assay in white blood cells from mice injected (ip) with 37.5 *μ*g/kg bw of MCLR, at several timepoints after treatment. Results are expressed as the mean percentage of DNA in comet tails (±SD) of 6 animals per treatment condition. The positive control, ethylnitrosourea (ENU) (100 mg/Kg), induced a 2- to 6-fold increase in the percentage of tail DNA. ∗represents a statistically significant difference between the treated and the control cells (*P* < 0.05).

The results from the comet assay in mice white blood cells (Figure 4) showed that MCLR exposure yielded a rapid and transient 2-fold increase in the percentage of DNA in tail, 30 min after treatment (*P* = 0.041). At the other posttreatment timepoints, the level of DNA damage was similar to that observed before MCLR administration.

**Figure 5:**
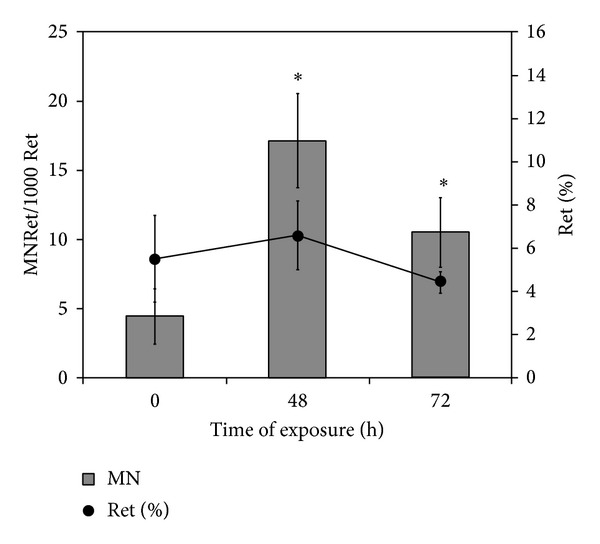
Results of the micronucleus assay in reticulocytes (Ret) from mice injected (i.p.) with 37.5 *μ*g/kg bw of MCLR. Results are expressed as the mean frequency of micronucleated reticulocytes (MNRet) (±SD) from 6 animals at several timepoints (primary axis). The negative control consisted of samples collected at 0 h. The positive control, ethylnitrosourea (100 mg/Kg), stimulated the increase of MNRet after 48 h (13-fold) and 72 h (2-fold) of exposure. In the secondary axis the percentage of reticulocytes was included. ∗represents a statistically significant difference between the treated and the control cells (*P* < 0.05).

As for the micronuclei data, mice exposure to MCLR caused a significant 3.9- and 2.3-fold increase in the frequency of MNRet at 48 h (*P* < 0.0001) and 72 h (*P* = 0.003) after treatment, respectively (Figure 5). There were no significant differences between the percentage of Ret in MCLR-treated and control samples.

## 4. Discussion

In the present work, we combined the micronucleus/FISH and the comet assays to characterize the potential genotoxicity of MCLR and its mechanisms on distinct* in vitro* and* in vivo* biological models: permanent cell lines representative of liver and kidney and mouse blood cells.

The results from the MN assay show that noncytotoxic concentrations (5 and 20 *μ*M, 24 h) of MCLR are able to significantly increase the frequency of MN, in both Vero-E6 and HepG2 cells, suggesting a capacity to induce permanent chromosome damage, either chromosome fragmentation or aneuploidy. Another study had previously reported MCLR-induced MN in a human T-cell leukemogenic line, the TK6 cell line [37], but a similar dose-range exposure (between 1 and 40 *μ*M of MCLR) did not induce MN neither in CHO-K1 cells [38, 39] nor in human lymphocytes, although they seem to be able to uptake the toxin [56].

In order to disclose the mechanism behind the MN formation (clastogenesis or aneugenesis) the content of MCLR-induced MN was characterized in HepG2 cells. Among the MN detected in unexposed cells, a predominance of MN containing whole chromosomes or centric fragments was found with a ratio of 5 : 1 cm+ : cm− MN. The majority of MN observed following MCLR treatment was also cm+ but a ratio of 3 : 1 cm+ : cm− MN was determined, indicating that MCLR induced more cm− than cm+ MN. These data suggest that MCLR is able to act through clastogenic and aneugenic mechanisms, with some predominance of clastogenesis. Given that the formation of MCLR-DNA adducts has never been demonstrated [35] the clastogenic effect of MCLR may be mediated by chromosome breaks indirectly generated by oxidative DNA adducts. Even though these have been previously reported in rat liver cells [35, 36], we did not detect any induction of oxidative DNA damage by the FPG-modified comet assay in MCLR-treated Vero-E6 cells. On the other hand, it is more plausible that MCLR can act by an aneugenic mechanism through the indirect disturbance of the mitotic spindle, caused by its well-documented inhibitory activity of protein phosphatases PP1/PP2A [57, 58]. It is known that PP1/PP2A, through their central role on phosphorylation-dephosphorylation reactions, participate in the control of assembly and constant turnover of microtubules [59], required for both spindle formation and chromosome movement [60]. Disturbance of the mitotic spindle by MCLR (≥50 *μ*M) was, in fact, demonstrated for CHO-K1 cells [61]. Additionally, its effect on cytoskeleton components is well known [62–66] and we have also observed MCLR-induced morphological and ultrastructural changes (disassembly, depolymerization, and aggregation) in microfilaments and microtubules of Vero-E6 cells [67] at the same dose range as that found to cause micronucleus formation in Vero-E6 and HepG2 cell lines.

The results from the comet assay show that MCLR does not induce DNA damage in Vero-E6 cells, independently of the timepoints tested. Furthermore, no oxidative DNA damage was detected, as revealed by the DNA repair enzyme FPG, which converts oxidised purines, including the major purine oxidation product 8-oxo-dG, into single-strand breaks (therefore detectable by the comet assay) through base excision [68]. The negative results disagree with previous reports from Žegura et al. [30–32], describing a dose-dependent and transient induction of DNA damage in MCLR-treated HepG2 and Caco-2 cells but are in line with the negative data reported for other cell lines (human astrocytoma IPDDC-A2 and human B-lymphoblastoid NCNC cells) [33]. Nong et al. [29] have also reported positive results in HepG2 cells exposed to MCLR, but for a higher toxin doserange (30–100 *μ*M), raising the question of a possible confounding effect from DNA degradation due to early apoptosis. A similar conclusion came out from a study that found a positive correlation between the frequency of apoptotic cells and the level of DNA damage as assessed by the comet assay, suggesting that MCLR-induced DNA damage could be related to the early stages of apoptosis rather than to genotoxicity [27].

Due to the uncertainties observed in the* in vitro* assays, we attempted to clarify whether mice exposure to MCLR was able to produce DNA and chromosome damage in peripheral blood cells. Our results from the comet assay in leukocytes showed that MCLR (37.5 *μ*g MCLR/kg bw, i.p. route) induced a 2-fold transient increase in the level of DNA breaks, 30 min after exposure. This suggests that although MCLR was able to induce DNA damage leukocytes might have had the ability to rapidly repair those lesions, in accordance with a previous report on primary cultured rat hepatocytes [36]. In that study, MCLR (≤10 ng/mL) induced the transient formation of 8-oxo-dG, peaking at 6 h after treatment and then declining, suggesting a fast repair of the DNA lesions [36]. However, two other studies reported that microcystins failed to induce DNA damage (evaluated by the comet assay) in rodent's blood cells [10, 69]. For example, in male Fisher F344 rats repeatedly exposed for 30 days to 10 *μ*g MCYR/kg bw (administered at every second day), no effect was detected in lymphocytes and spleen cells although a significant increase of tail DNA was observed in liver, kidney, and brain cells [69]. A study from Gaudin et al. [10] with female Swiss albino mice administered at 3 and 24 h with 10–50 *μ*g MCLR/Kg bw (i.p. route) showed no induction of DNA damage in blood cells. All these results can hardly be compared given the differences between the exposure conditions to microcystins and, putatively, differences in the toxin bioavailability. However, we can hypothesize that, in those two previous studies, a DNA damaging effect might have occurred, although it had been repaired prior to the genotoxicity analysis by the comet assay. Concerning the MN assay, we observed that 48 h and 72 h following i.p. injection of the toxin, the frequency of MNRet was significantly raised as compared to the frequency determined before injection. This corroborates the report of Ding et al. [28] who also observed MN induction in reticulocytes from male Kunming mice injected twice (i.p., interval of 24 h) with cyanobacterial extracts containing MCLR (0.45–45 *μ*g MCLR/kg bw). Our results confirm that MCLR is genotoxic, through its ability to cause genetic instability in the precursors of erythrocytes present in the bone marrow.

Taken together, both* in vitro* and* in vivo* data suggest that the MN assay can be more sensitive than the comet assay to reveal the genotoxicity of MCLR, which is probably an indirect genotoxicant. Although it is far from being consensual, this observation agrees with some studies that compared the two methods to detect the genotoxicity of several organic compounds in HepG2 cells [46] and of a complex environmental mixture [50].

In summary, using kidney- and liver-derived cell lines and blood cells from MCLR-exposed mice, we showed that MCLR consistently induces structural and numerical chromosome alterations, although we could not observe any consistent increase neither in the level of DNA damage nor in that of oxidative DNA damage, using the comet assay. Aneuploidy induction might be a common mechanism of MCLR genotoxicity in liver, kidney, and bone marrow cells and might result from its interference with the mitotic spindle as part of a more general effect on cell cytoskeleton, while clastogenesis could result from an indirect mode of action. Both chromosome breakage and chromosome loss have been associated with cell transformation towards malignancy and human cancer development [70] and thus can contribute to the carcinogenic activity of MCLR.

## Figures and Tables

**Table 1:** Results of DNA damage assessed by the comet assay (% of DNA in tail) in MCLR-treated Vero-E6 cells.

MCLR (*μ*M)	% of DNA in the comet tail			
	Exposure time			
	4 h		24 h	
	Buffer	FPG	Buffer	FPG
0	4.11 ± 0.21	5.13 ± 0.28	3.29 ± 0.01	3.76 ± 0.42
5	4.21 ± 0.38	4.96 ± 0.42	3.16 ± 0.39	3.37 ± 0.14
10	3.82 ± 0.04	6.41 ± 0.55	2.74 ± 0.44	4.08 ± 0.21
20	4.77 ± 0.50	5.74 ± 0.75	3.67 ± 0.16	4.22 ± 0.14

Values are the mean (±SE) of three independent experiments. The percentage of DNA in the comet tail induced by the positive control (H_2_O_2_, 30 min) was 13.73 ± 0.62 and 22.86 ± 0.94, without and with FPG treatment, respectively.
